# Anxiety, depression and quality of life in individuals with phantom limb pain

**DOI:** 10.1590/1413-78522015230200990

**Published:** 2015

**Authors:** Mariana Theozzo Padovani, Marielza Regina Ismael Martins, Alexandre Venâncio, José Eduardo Nogueira Forni

**Affiliations:** 1 Faculdade de Medicina de São José do Rio Preto, São José do Rio Preto, SP, Brazil, 1. Faculdade de Medicina de São José do Rio Preto, São José do Rio Preto, SP, Brazil; 2 Hospital de Câncer de Barretos, Barretos, SP, Brazil, 2. Hospital de Câncer de Barretos, Barretos, SP, Brazil

**Keywords:** Amputation, Chronic pain, Quality of life

## Abstract

**OBJECTIVE:** This study aims to evaluate emotional factors such as anxiety and depression, and the Quality of Life of individuals with chronic persistent pain after amputation in order to identify the interindividual variation in response to pain. **METHODS:** This was a descriptive, exploratory and cross-sectional study with quantitative approach. Twenty seven patients were interviewed. The instruments have rated the sociodemographic, clinical and economic profile (semistructured interview) and the Quality of Life (generic Quality of Life questionnaire SF-12) and emotional factors (HAD scale) of the interviewed patients. **RESULTS:** It was identified that the most frequent amputations occur in males aged 18-38 years and are related to occupational accidents. The Quality of Life was compromised in both components of physical and mental health. Furthermore, anxiety levels were more prevalent in the range from aged 18 to 38 years old, while the levels of depression were most prevalent among the elderly (60 to 80 years old). **CONCLUSION:** The impairment of Quality of Life and change in the perception of body image has a major impact on adherence to the rehabilitation program and the functional prognosis. Therapeutic orientation is, therefore, critical after this type of surgery. **Level of Evidence II, Descriptive and Exploratory Study.**

## INTRODUCTION

Amputations are characterized by the removal of an organ/limb or part thereof located at the end of the human body.^1 ^Etiologies leading to amputation are various and can be divided into vascular causes, such as diabetes mellitus, atherosclerosis and vasculitis, as well as nonvascular causes as trauma, neoplasia, burns, congenital or infectious conditions.^2^ According to the survey of lower limbs amputees treated at Lar Escola São Francisco in São Paulo, SP, Brazil, from 2006 to 2012, the main reasons were vascular causes amputations.^3^

This type of surgical procedure aims to consider what it is available to replace the function of that limb, preferably to its aesthetics. However, amputation and any other surgical procedure can lead to complications, such as bleeding, infection, skin tear, joint contracture and especially phantom limb pain. Furthermore, we must consider the psychological trauma since the patients are faced with a situation that involves a change in their locomotion, work and social living.^4^

Phantom limb pain (PLP) refers to the sensation of pain in the missing part of the amputated limb, presenting in different ways such as a burning sensation, a grip, or a pain which may vary in intensity and frequency.^5^ For a long time, it was believed that the origin of the phantom sensation was psychic. However, it is known today that this phenomenon is also physiologically related.^6^

There are two theories that explain its pathophysiology. The first considers that this pain is a phenomenon that occurs from top to bottom, i.e. it is triggered by the reorganization of the mapping of structures depicted in the cerebral cortex, in a sensory and motor plasticity process. The second considers that this phenomenon occurs from the bottom up, i.e. it occurs by excessive stimulation and not by its loss, ectopically generated in the primary afferent neurons in the dorsal root ganglion, that before enervated the amputated limb. Among these theories, the first is the most widely accepted.^7^

This kind of pain occurs in 50-80% of amputation cases and can occur days after surgery for reasons not yet fully understood.^8^ There are factors that may aggravate the pain, among them attention, emotion, touching the stump or pressure, temperature change, autonomous reflexes, pain from other sources, placement of a prosthesis. However, factors that relieve pain include resting, distraction, stump movement, wearing a prosthesis, lifting the stump, percussion or stump massage.^9^

It should be noted that phantom limb pain differs from pain in the stump, or residual limb, which is due to skin complications, vascular compromise, inappropriate healing, painful neuromas, excess soft tissue and bone irregularities.^10^ Both types of pain can interfere with the physical and psychosocial rehabilitation of the amputee, compromising the acquisition of skills and quality of life.^11^

The impact of amputation in the experience of any individual translates into changes in his body image, beyond that, it infers on the psychosocial adjustment of individuals who experience the phenomenon, with influence on their self-esteem and the possible emergence of psychopathological symptoms and on social functioning.^12^

Within this context, this study aims to assess emotional factors such as anxiety and depression and quality of life of individuals with post-amputation persistent chronic pain in order to identify the interindividual variation in response to pain and contribute to knowledge and practice of health professionals involved in assisting this population.

## MATERIALS AND METHODS

This is a descriptive, exploratory, cross-sectional study with a quantitative approach. The research project was approved by the Research Ethics Committee of FAMERP (2384/2010) and held at the Pain Clinic Outpatient and Orthopedics and Traumatology Service, *Hospital de Base* (FUNFARME/FAMERP). Inclusion criteria were patients of both genders aged over 18 years old that have had persistent pain postoperatively to independent amputation whichever body part or level, with cognitive level sufficient to understand the assessments and consent to participate in the study by signing the Post-Informed Consent Term. 

The evaluation of the subjects was performed using primarily a semi-structured interview with sociodemographic, economic and clinical characteristics (marital status, schooling, age, gender, occupation, housing and family relationships, besides questions about the use of alcohol and tobacco and related diseases), followed by a characterization instrument regarding the amputation surgery (level of amputation, cause leading to surgery and the time of prosthesis use, if it applied). Subsequently an instrument for the analysis of daily activities and practice was applied (sleeping, appetite, deambulation, home activities, work, personal hygiene, bowel movements, interpersonal relationships, concentration, sexual activity, mood and leisure) which were marked with the corresponding values: (1) No alteration (2) Partially compromised. (3) Fully compromised (4) does not apply. Pain was assessed using a visual analog scale (VAS),^13^ which measures the intensity of pain in patients where zero means total absence of pain and 10 maximum pain. Emotional factors such as anxiety and depression were assessed using the HAD scale (Hospital Anxiety and Depression Scale),^14 ^which contains 14 multiple-choice questions, seven for anxiety and seven for depression, which has the cut-off point at eight for anxiety and nine for depression. The quality of life was assessed by the generic questionnaire on quality of life SF-12^15^ consisting of 12 questions that address the physical component (functional capacity and limitation due to physical aspects) and the mental component (pain, vitality, social aspects, limitation due to emotional aspects and mental health). All subjects of both groups were subjected to a single assessment.

The descriptive analysis was performed in the Excel software. Qualitative data were analyzed by odds ratio and ordinals by nonparametric tests. All statistical analysis was performed using significance level at 0:05.

## RESULTS

Twenty-seven patients from the Pain Clinic Outpatient and Orthopedics and Traumatology Service, *Hospital de Base* (FUNFARME/FAMERP) participated in this study, 16 of them being male (59%) and 11 female (41%). Among the patients, 38% (n=10) were young adults (from 18 to 38 years old), 33% (n=9) were elderly (60-80 years old) and 29% (n=8) were in the range 39-59 years old. Mean education was 6.5±3.0 years. Of the participants 48% (n=13) were married, 22% (n=6) were single, 22% were widowed and 8% (n=2) divorced. Most were still working (n=15/55%).

As for the self perception of the familiar aspects 77% (n=21) believed they have a good relationship with their family, while 9% (n=2) considered having a bad relationship. Among the subjects evaluated 22% (n=6) were smokers and 18% (n=4) were drinkers. The main diseases that have been reported are associated with diabetes mellitus and hypertension (both with n=3/11%). Other diseases reported were cirrhosis (n=1/3%) and chronic renal failure (n=1/3%). Most respondents own their house (n=13/44%), while 40% (n=12) pay rent and 6% (n=2) live with relatives. Sociodemographic and clinical characteristics are summarized in Table 1.

**Tabela 1.** Sociodemographic and clinical characteristics of the studied sample (n=27).

Variables	%	Mean + SD	p
Gender			
Masculine	59 (n=16)		0.048*
Feminine	41 (n= 11)		
Age (years old)			
18-38	38 (n=10)	45.6 ± 17.2	0.056
39 - 59	29 (n=8)		
60 - 80	33 (n=9)		
Schooling (years)		6.5 ± 3.0	
Marital status			
Married	48 (n=13)		0.028*
Single	22 (n=6)		
Widow	22 (n=6)		
Divorced	8 (n=2)		
Occupation			
Works	55 (n=15)		0.056
Does not work	45 (n=12)		
Self perception of familiar aspects			
Good	77 (n=21)		0.028*
Moderate	14 (n=4)		
Bad	9 (n=2)		
Smoker	22 (n=6)		0.065
Drinker	18 (n=4)		
Associated diseases			
Diabetes	11 (n=3)		0.065
Hypertension	11 (n=3)		
Cirrhosis	3 (n=1)		
Cirrhosis	3 (n=1)		
Chronic renal failure	3 (n=1)		
Housing			
Own	44 (n= 12)		0.058
Rented	40 (n=13)		
Lives with relatives	6 (n=2)		

Chi-square test *p< 0.05: statistically significant difference.

Chi square test - * p <0.05: significant statistical difference.

Regarding daily activities 44.4% (n=12) of subjects reported sleeping was partially compromised after amputation surgery, while 25.9% (n = 7) reported no change in this regard. Regarding appetite mostly (n=19 / 70.3%) reported no change and only 7.4% (n=2) of patients reported to have partially compromised appetite. As for deambulation 40.7% (n=11) of patients reported partial impairment, 14.8% (n=4) of patients reported total impairment and 25.9% (n=7) had no effect in this regard. One third (33.3%, n=9) of the subjects reported no changes in household activities, while 29.6% (n=8) reported partial impairment of these activities and 11.1% (n=3) reported total impairment. 

Regarding work limitation was greater, 29.6% (n=8) of patients were totally impaired in this regard, 25.9% (n=7) had milder impairment and only 22.2% (n=6) were not impaired at all. As for personal hygiene 29.6% (n=8) of the subjects reported having partial impairment, while 44.4% (n=12) reported no change. Fourteen point eight percent (n=4) of the subjects reported total impairment of bowel habits, 22.2% (n=6) reported partial impairment and 33.3% (n=9) had no alteration whatsoever. Interpersonal relationships were partially deteriorated to 29.6% (n=8) of individuals, totally deteriorated to 11.1% (n=3) and not affected to 40.7% (n=11) of them.

**Figura 1.**
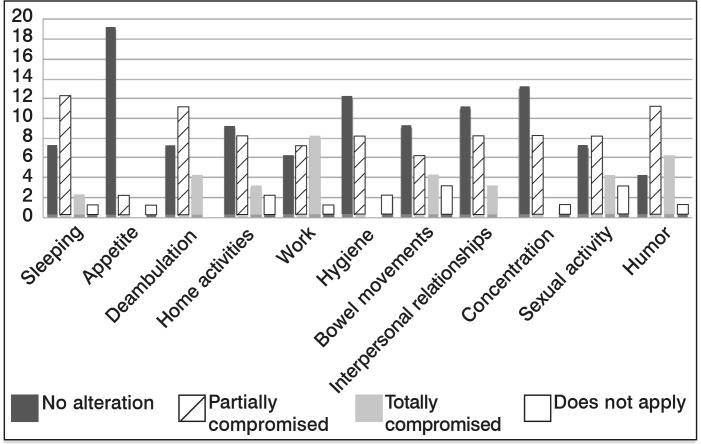
Data on activities of practical daily life on the sample studied (n=27).

Concentration was abnormal in 29.6% (n=8) of patients and in 48.1% (n=13) it was not altered. Regarding sexual activity 29.6% (n=8) of respondents reported partial impairment, 14.8% (n=4) reported total impairment and 25.9% (n=7) reported no alteration. Humor was the aspect that was most altered, since 40.7% (n=11) evaluated the reported partial impairment, 22.2% (n=6) reported total impairment and only 14.8% (n=4) reported no change at all. (Figure 1)

Regarding the pain sensation, burning was the most common type (42% - n=11) followed by "needle type" (36% - n=9) and burning feeling (22% - n=7). Only seven patients (25%) and wore prosthesis. Regarding medication adherence, 51% (n=14) used medications such as anticonvulsants, antidepressants and anesthetic blocks. Thirteen patients (49%) reported use of anti-inflammatory or analgesic drugs, without improvement. There were no reports of use of regular or intermittent analgesia prior to amputation. Rehabilitation was done in 70% (n=18) with orientation such as stump lifting, percussion and massage. (Table 2)

**Tabela 2.** Data referring to pain levels, cause a

	%	Mean ± SD
Amputation level		
Transfemoral	44 (n=12)	
Transtibial	11(n=3)	
Transcarpian	25(n=7)	
Transhumeral	20(n=5)	
Pain - VAS		
Low (0-2)	25(n=7)	6.4 ±1.5
Moderated (3-7)	44(n=12)	
Intense (8-10)	29(n=8)	
Amputation time (months)		27.2 ±12.0
Causes		
Work injury	37(n=10)	
Trauma	18(n=5)	
Trombose	18(n=5)	
Cancer	7(n=2)	
Diabetes	20(n=5)	
Rehabilitation time (months)		11.5 ± 3.0

**Figura 2.**
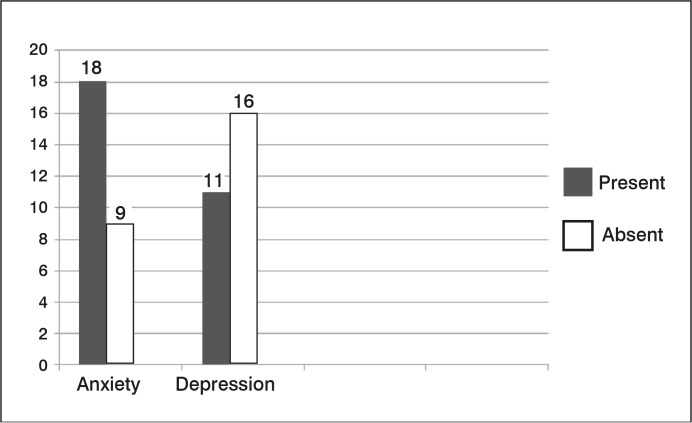
Number of patients by level of severity of depression symptoms and indication of average grades (n=27).

Regarding anxiety and depression the number of patients ranked by the level of severity of symptoms of depression with their mean scores on the Hospital Anxiety and Depression Scale (HAD) are shown in Figure 2.

It has been noted that anxiety levels were higher (18/27) than depression (11/27), and the quality of life was compromised in both components (<50), where the physical health component was 42 and the mental health component 40.

**Figura 3.**
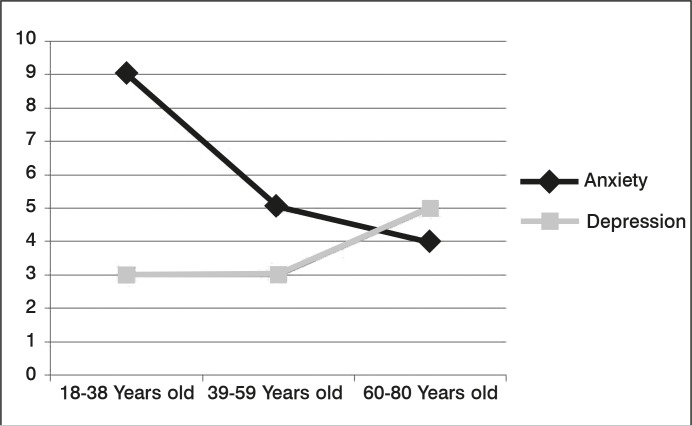
Association between age of individuals in sample and presence of anxiety and depression (n=27).

The study also included associations between the individual's age and the presence of anxiety and depression. (Figure 3)

Importantly, the anxiety levels were more prevalent in the age group of 18-38 years old, while the levels of depression were more prevalent among the elderly (60-80 years old).

## DISCUSSION

This study investigated the sociodemographic and clinical characteristics of patients from Pain Clinic Outpatient and Orthopedics and Traumatology Service, *Hospital de Base* (FUNFARME/FAMERP) who presented phantom limb pain after amputation surgery, as well as the impairment of daily activities and quality of life of these individuals, besides depressive and anxiety symptoms.

The loss of a limb disrupts the integrity of the body and affects physical and psychological conditions of patients who undergo amputation surgery. This type of surgery means a huge impact, not only for the patient's body and the way he notices it, as well as the perception of the environment around him.^16^ In view of this problem, this cross-sectional study aims to evaluate the quality of life, anxiety and depression in patients with phantom limb pain after amputation surgery, since in Brazil recent studies on this subject are scarce.

Compared to the study of Resende *et al*.^17^ sociodemographic data were very similar, since in the cited study 21 individuals aged 20-69 years old, mean age of 42.62 years old with limb amputation were evaluated, while the present study evaluated 27 individuals between 18 and 80 years old with a mean age of 45.6 years old. In both most individuals were male (71.4% and 59%). In that study^17^ the majority of respondents were married (47.6%), as well as in the present study (48%). In both studies, most patients remain employed and in the cited study 71.4% had their own income, as compared with 55% in this study. 

Regarding to clinical aspects, in both studies, the major cause of amputation were accidents (81% and 37%). However, most of amputations in this study was of the lower limbs (55%), while in the study of Resende *et al*.^17^ it was the upper limbs (67.3%), likewise, most patients did not use prosthesis (71.4% and 75%). In the present study, only seven individuals (25%) wore prosthesis.

As to daily living activities, this study reveals that most of them are partially or totally compromised, except appetite. Activities such as working and humor proved to be the most affected. According to the study of Batista *et a*l.^18^ this compromise occurs due to the experience of a daily life permeated by difficulties, limitations and restrictions imposed by amputation; suffering by dependence on others, personal and economic limitations or inadequacy of public policies, besides the personal and professional life changed by the surgery and living in fear of loss of physical integrity.

The phantom limb pain it is a type of chronic post-surgical pain, whose incidence is high and that affects qualitatively the patients' lives, though often neglected by the medical team. It is a morbidity difficult to treat, and prevention is the most effective measure.^19^ It is, therefore, extremely important to have a larger number of studies related to this topic, since it adversely affects a large proportion of amputees, besides, their mechanisms are complex and not yet fully understood.

This type of pain is manifested in various ways, it may be characterized as mild to severe, intermittent or constant, like a stab, a shot, a tingle, a colic, a punch by a needled, and/or a burning feeling.^8^ The most reported type of pain in this study is the burn type (42% - n=11) followed by needle punch (36% - n=9) and tingle (22% - n=7). Most of patients (44%) reported a moderate pain intensity.

The results of the study indicate that patients with phantom limb pain have a lower quality of life, mainly related to the impairment of daily activities and an increase in anxiety levels, especially among young people (18-38 years) and depression in the elderly (60-80 years old). In the study by Vaz *et al*.^20^ the results were consistent, since the evaluated the sample in that study also showed a high prevalence of depression/anxiety symptoms.

## CONCLUSION

As well as the perception of body image, phantom limb pain leads to changes in the psychosocial profile, which result in a major impact on adherence to the rehabilitation program, the functional outcome and quality of life. Therefore, its evaluation and therapeutic management should be part of the approach of patients undergoing amputation surgery, giving the importance this type of clinical condition deserves to have.
